# Use of complementary and integrative medicine for mental disorders–a cross-sectional study in Germany

**DOI:** 10.3389/fmed.2026.1882619

**Published:** 2026-08-19

**Authors:** Julia Siewert, Hannah Wackermann, Katja Icke, Nicolas Volz, Michael Teut, Benno Brinkhaus

**Affiliations:** 1 Institute of Social Medicine, Epidemiology and Health Economics, Charité – University Medical Center Berlin, Corporate Member of Freie Universität Berlin and Humboldt-Universität zu Berlin, Berlin, Germany; 2 Institute of Social Medicine and Epidemiology, The Brandenburg Medical School Theodor Fontane, Brandenburg/Neuruppin, Germany

**Keywords:** complementary and integrative medicine, mental disorders, psychiatry, prevalence, disclosure, Germany

## Abstract

**Background:**

Mental disorders pose a substantial global health burden. Complementary and Integrative Medicine (CIM) is widely used; however, data on CIM use in mental disorders are limited. The aim was to investigate CIM prevalence, usage patterns, disclosure, and perceived benefits among individuals with mental disorders in Germany.

**Methods:**

A cross-sectional study was conducted among adults with psychiatric disorders in Germany between June 2023 and August 2024. Participants were recruited through a nationwide anonymous online survey and on-site surveys in randomly selected outpatient psychiatric, psychosomatic, and psychotherapeutic settings in Berlin and Brandenburg. The 33-item questionnaire assessed lifetime and current CIM use, diagnostic and sociodemographic characteristics, physician disclosure, recommendation sources, and perceived effectiveness. Associations were analysed using logistic regression.

**Results:**

Seven hundred sixty-eight participants (mean age 43.2 years; 77.3% female) completed the questionnaire. Lifetime CIM use was 92.3, and 68.1% reported current use. The most commonly used approaches (lifetime use) were meditation (63.9%), yoga (60.8%), and dietary supplements (59.1%); positive experiences were highest for meditation (76.9%) and yoga (74.6%). Among CIM users, 60.9% informed their physician. Physician awareness was associated with more favourable CIM evaluations (adjusted OR 2.69, 95% CI 1.63–4.49). Recommendations were mainly based on self-directed research (59.9%) and friends’ advice (31.2%).

**Conclusion:**

CIM use among individuals with mental disorders is highly prevalent and predominantly self-initiated. However, many patients do not disclose their CIM use to clinicians. Physician awareness of patients’ CIM use was associated with more positive patient-reported evaluations, suggesting that proactive enquiry and open communication about CIM use may facilitate safer and more coordinated care. Further clinical research is required to evaluate the effectiveness, safety, and potential interactions of commonly used CIM approaches in psychiatric care.

## Introduction

Psychiatric disorders impose a *global burden*, leading to reduced quality of life, disability, and health-care costs. World Health Organization (WHO) estimates indicate that over one billion people worldwide live with mental health conditions (1). In 2021, approximately 444,397,716 new cases and 155,418,119 disability-adjusted life years (DALYs) worldwide were attributable to mental disorders (2). From 1990 to 2021, age-standardised incidence and DALY rates increased significantly, exacerbated by the COVID-19 pandemic (2). Major gaps persist in global mental health data collection, particularly in low-income countries lacking reliable prevalence estimates (3).

In Germany, nationwide outpatient claims data from statutory health insurers indicate that the diagnostic prevalence of mental disorders rose from 33.4 to 37.9% between 2012 and 2022, with marked increases in depression, anxiety, substance-related disorders, and PTSD, and the greatest growth among adolescents and older adults (4). Survey data in Germany show that the share of adults above the cutoff for depressive symptoms nearly doubled from 7.5% in 2020 to 14.8% in 2023 (5). Despite the availability of evidence-based conventional treatments, some patients continue to face challenges in achieving satisfactory treatment outcomes. Not all patients experience satisfactory treatment benefits (6–10), and some experience treatment-related adverse effects. Common treatment-related adverse effects include weight gain, sexual dysfunction, sedation, and gastrointestinal symptoms (11–13). These adverse effects may reduce adherence (14) and prompt some patients to consider CIM approaches. Structural challenges—including long waiting times for psychotherapy, limited availability of specialized services, and fragmented care pathways (15–17)—may further increase patients’ interest in CIM treatments. Complementary and integrative medicine (CIM), as defined by the World Health Organization (WHO), refers to health care practices that are not part of mainstream medicine and are used alongside conventional care to support health and well-being. CIM includes a broad range of approaches such as herbal medicine, nutritional supplements, mindfulness-based interventions, meditation, yoga, and acupuncture (18).

Taken together, international evidence suggests that patients with psychiatric disorders often use CIM alongside conventional treatment to address unmet needs not fully covered by standard care (19–22). Reported reasons for CIM use include seeking additional symptom relief, dissatisfaction with limitations of conventional treatments, and preferences for more holistic treatment approaches that align with patients’ values and beliefs (19, 21, 23). These factors contribute to the growing relevance of CIM in mental health care worldwide, with cross-national surveys indicating widespread use among individuals with psychiatric disorders (22).

Importantly, CIM is commonly used as an adjunct to conventional psychiatric treatment rather than as a replacement, although utilisation patterns vary across health care systems and treatment contexts (21, 23–25).

In Germany, representative survey data from the general population indicate substantial CIM use, with 32% of adults reporting CIM use within the last 12 months and 70% reporting lifetime use (26).

Although evidence from psychiatric populations remains limited, findings from broader non-psychiatric health care settings suggest that disclosure of CIM use to treating clinicians is often inconsistent, with many patients not routinely informing health care providers about CIM use (27, 28). This may have implications for treatment safety, care coordination, and communication between patients and health care providers.

Despite the growing clinical relevance of CIM, data on the prevalence, usage patterns, and determinants of CIM use in psychiatric disorders remain scarce, particularly in Germany.

This study aimed to investigate the prevalence and usage patterns of CIM in psychiatric disorders in Germany, including clinical and sociodemographic characteristics, disclosure practices, sources of recommendation, and perceived treatment benefits.

## Methods

### Study design and setting

The PSYKIM study (Utilization of Complementary and Integrative Therapies in Outpatient Mental Health Care in Germany) was a cross-sectional survey assessing CIM use among adults with psychiatric disorders in Germany. Ethical approval was obtained from the Charité ethics committee (EA2/058/23), and the study was registered in the German Clinical Trials Registry (DRKS00032426).

All procedures complied with the Declaration of Helsinki and Good Clinical Practice (ICH-GCP) guidelines. The study was coordinated by the Institute of Social Medicine, Epidemiology and Health Economics, Charité – Universitätsmedizin Berlin. Data collection was anonymous. A qualitative PSYKIM substudy is reported elsewhere (29). Analyses in this manuscript focus on sample characteristics, use of conventional medicines and CIM, perceived effectiveness of CIM, patterns of CIM use across primary psychiatric disorders, physician awareness and sources of recommendation. Additional analyses (e.g., EQ-5D, health-related quality of life) will be reported separately.

### Participants and eligibility criteria

Adults with psychiatric disorders were recruited from ambulatory psychiatric, psychosomatic, and psychotherapeutic care settings in urban and rural areas of Berlin and Brandenburg, and through a nationwide anonymous online survey.

Recruitment used two strategies: (1) a nationwide online survey disseminated via mental health organisations, self-help groups, and university networks across Germany; and (2) an on-site survey in psychiatric and psychotherapeutic outpatient facilities in Berlin and Brandenburg randomly selected from the regional Association of Statutory Health Insurance Physicians (Kassenärztliche Vereinigung) registry.

All participants received written study information and provided informed consent.

Combined recruitment aimed to capture a broad range of ambulatory psychiatric care users across care settings and levels of digital literacy.

Eligible participants were adults aged ≥18 years who reported having received a psychiatric diagnosis from a physician or psychotherapist (acute, chronic, recurrent, or remitted). Inclusion criteria were sufficient German proficiency and cognitive ability to complete a ~ 20-min self-administered questionnaire.

Exclusion criteria included current inpatient treatment, insufficient capacity to provide informed consent, insufficient German proficiency, or anticipated non-compliance (e.g., unwillingness to complete the questionnaire).

Participants with mild cognitive limitations or limited digital literacy received support from trained study staff during on-site data collection.

### Survey instrument

This study defined CIM according to the World Health Organization (WHO) as culturally rooted knowledge, skills and practices used to maintain health or prevent, diagnose and treat physical or psychiatric disorders outside medicine (30). Accordingly, CIM approaches were defined as health care approaches originating outside conventional medicine, including traditional, complementary and integrative practices. In this survey, assessed CIM modalities included acupuncture, acupressure, Tai Chi/Qi Gong, traditional Chinese medicine, osteopathy, chiropractic, manual therapy, herbal medicinal products, nutritional supplements, dietary modification, hypnosis, yoga, meditation, homeopathy, Ayurveda, anthroposophic medicine, and other self-reported complementary treatments. Psychoactive substances were not assessed as separate CIM categories but could be reported in free-text responses.

The CIM section specifically assessed the use of these approaches in relation to participants’ psychiatric symptoms or psychological complaints, rather than general CIM use. Participants were asked whether they had ever used each CIM modality against their psychological complaints and whether it was currently used or had been used within the previous 12 months.

The structured questionnaire was developed by the study team at the Institute of Social Medicine, Epidemiology and Health Economics, Charité – Universitätsmedizin Berlin, based on national and international surveys of complementary medicine use and adapted to the psychiatric context. Content validity was ensured through expert consensus among psychiatrists, psychotherapists, physicians specialised in complementary and integrative medicine, as well as experts in naturopathic and traditional Chinese medicine.

The questionnaire covered the following domains: sociodemographic characteristics; psychiatric diagnoses and symptom course; use of complementary and integrative medicine (type, frequency, and perceived effects); mode of use (self-initiated or physician-prescribed); disclosure to treating clinicians; economic aspects; and health-related quality of life (EQ-5D-5L) (Supplementary Material 1).

The instrument was administered in German and combined closed-ended multiple-choice and Likert-scale questions (5-point scales for perceived effectiveness and disclosure behaviour) with several open-text fields for qualitative comments.

In addition to predefined response options for CIM use, the questionnaire included a free-text field in which participants could report additional food supplements, herbal medicinal products or other complementary treatments used for their mental health condition. These responses were coded and analysed descriptively to capture treatments not covered by the structured items.

Perceived CIM effects were assessed based on participants’ subjective evaluation of whether each CIM approach improved their psychiatric symptoms or psychological complaints, using a standardised response format (complete improvement, partial improvement, no improvement, worsening, uncertainty).

Before field implementation, the questionnaire was pilot-tested with 15 psychiatric outpatients to assess clarity, comprehensibility and completion time. Minor wording adjustments were made following feedback to improve readability and reduce ambiguity.

### Data collection and management

At on-site locations, participants completed the questionnaire on paper, on a tablet provided by study staff, or on their own device via a secure survey link. Trained assistants supported recruitment and on-site participation.

Responses were collected via the SoSci Survey platform (SoSci Survey GmbH, Munich, Germany) hosted on secure Charité servers, and were transmitted through encrypted connections and anonymised upon entry. Data were stored on institutional project servers at the Institute of Social Medicine, Epidemiology and Health Economics and analysed in anonymised form.

Data handling complied with applicable data protection and institutional quality management regulations. Anonymised data were stored on secure institutional servers in accordance with data protection regulations. To encourage participation, respondents could enter a raffle for book vouchers.

### Statistical analysis

Data were analysed using IBM SPSS Statistics (version 29; IBM Corp., Armonk, NY, USA) and R (version 4.5.1; R Foundation for Statistical Computing, Vienna, Austria). All analyses followed a cross-sectional design. Prior to analysis, plausibility and completeness checks were performed to ensure data quality.

Descriptive statistics summarised study outcomes; continuous variables were reported as means with standard deviations and categorical variables as frequencies and percentages.

For analyses of perceived effects of individual CIM approaches, responses indicating complete or partial improvement were combined to represent perceived benefit.

Associations between variables were explored descriptively. Selected associations were examined using logistic regression models adjusted for age and sex, with results reported as adjusted odds ratios (aOR) and 95% confidence intervals.

## Results

### Sample characteristics

A total of 768 participants were included (77.3% female, 21.4% male, 1.3% diverse; mean age 43.2 years, SD 14.4) between June 2023 and August 2024. Participants were recruited in Berlin (30.5%), Brandenburg (31.5%) and other German federal states (38.0%).

Recruitment source was online for *n* = 340 participants and ambulatory/on-site for *n* = 426; data were missing for *n* = 2.

Most reported a recurrent course of illness (82.3%); 81.9% had experienced symptoms for more than 6 months and 26.6% were currently on sick leave due to psychiatric disorders. Educational background varied, including university education (32.2%), university of applied sciences (10.7%), and vocational education and training (21.7%).

Employment status varied: 44.1% were employed/civil servants, 6.6% self-employed, 9.3% unemployed, 8.7% students, 24.0% retired, 5.9% other and 1.4% pupils/in training. Data on monthly disposable income were provided by CIM users only. Among respondents, 28.2% reported €201–500, 19.1% €501–1,000, 12.1% €1,001–1,500 and 10.3% > €1,500; smaller proportions reported ≤€200. Most participants had statutory health insurance (92.4%), and 36.3% lived alone (Table 1).

**Table 1:** Sociodemographic characteristics.

Characteristic	*N* = 768
Sex	
Female	594 (77.3%)
Male	164 (21.4%)
Diverse	10 (1.3%)
Unknown	*n* = 0
Place of residence (Federal State)	
Berlin	234 (30.5%)
Brandenburg	241 (31.5%)
Other federal states	291 (38.0%)
Unknown	*n* = 2
Education	
Vocational apprenticeship	166 (21.7%)
Vocational school	71 (9.3%)
Technical school	65 (8.5%)
University of applied sciences	82 (10.7%)
University	246 (32.2%)
Other vocational qualification	19 (2.5%)
Currently in vocational training	48 (6.3%)
No vocational qualification	67 (8.8%)
Unknown	*n* = 4
Current employment	
Employed/Civil servant	338 (44.1%)
Self-employed	51 (6.6%)
Unemployed	71 (9.3%)
Studying	67 (8.7%)
Retired	184 (24.0%)
Other	45 (5.9%)
Pupil/In training	11 (1.4%)
Unknown	*n* = 1
Health insurance	
Private insurance	51 (6.7%)
Statutory insurance	708 (92.4%)
Other insurance	7 (0.9%)
No insurance	0 (0%)
Unknown	*n* = 2
People in household	
Living alone	278 (36.3%)
Living with others	487 (63.7%)
Unknown	*n* = 3
Age group	
18–29	146 (19.1%)
30–44	285 (37.2%)
45–59	204 (26.6%)
60+	131 (17.1%)
Unknown	*n* = 2
Currently on sick leave due to psychiatric disorders	
No	557 (73.4%)
Yes	202 (26.6%)
Unknown	*n* = 9
Currently retired early due to psychiatric disorders	
No	634 (83.0%)
Yes	130 (17.0%)
Unknown	*n* = 4
Age	43.2 (SD: 14.4)
Unknown	*n* = 2

The most frequently reported primary psychiatric disorders were depression (45.6%), stress-related disorders (including PTSD, adjustment disorder, and acute stress reaction; 16.2%), anxiety disorder (10.7%) and personality disorder (5.6%) (Supplementary Table 1). Comorbid psychiatric disorders were common: 64.7% reported additional psychiatric disorders, most frequently depression (41.5%), anxiety disorder (37.5%) and somatic symptom disorder (20.0%) (Supplementary Table 2). Use of apps or online therapies for the primary diagnosis was reported by 19.6%.

### Use of psychopharmacological medication

Overall, 61.6% reported psychopharmacological medication use. At the time of the survey, 40.8% were currently taking psychiatric medication, 14.1% had used it within the past 12 months and 20.9% previously. Free-text responses most commonly referenced psychopharmacological agents, particularly Escitalopram, Sertraline, Citalopram and Mirtazapine.

### Use of complementary and integrative medicine

**Figure 1:**
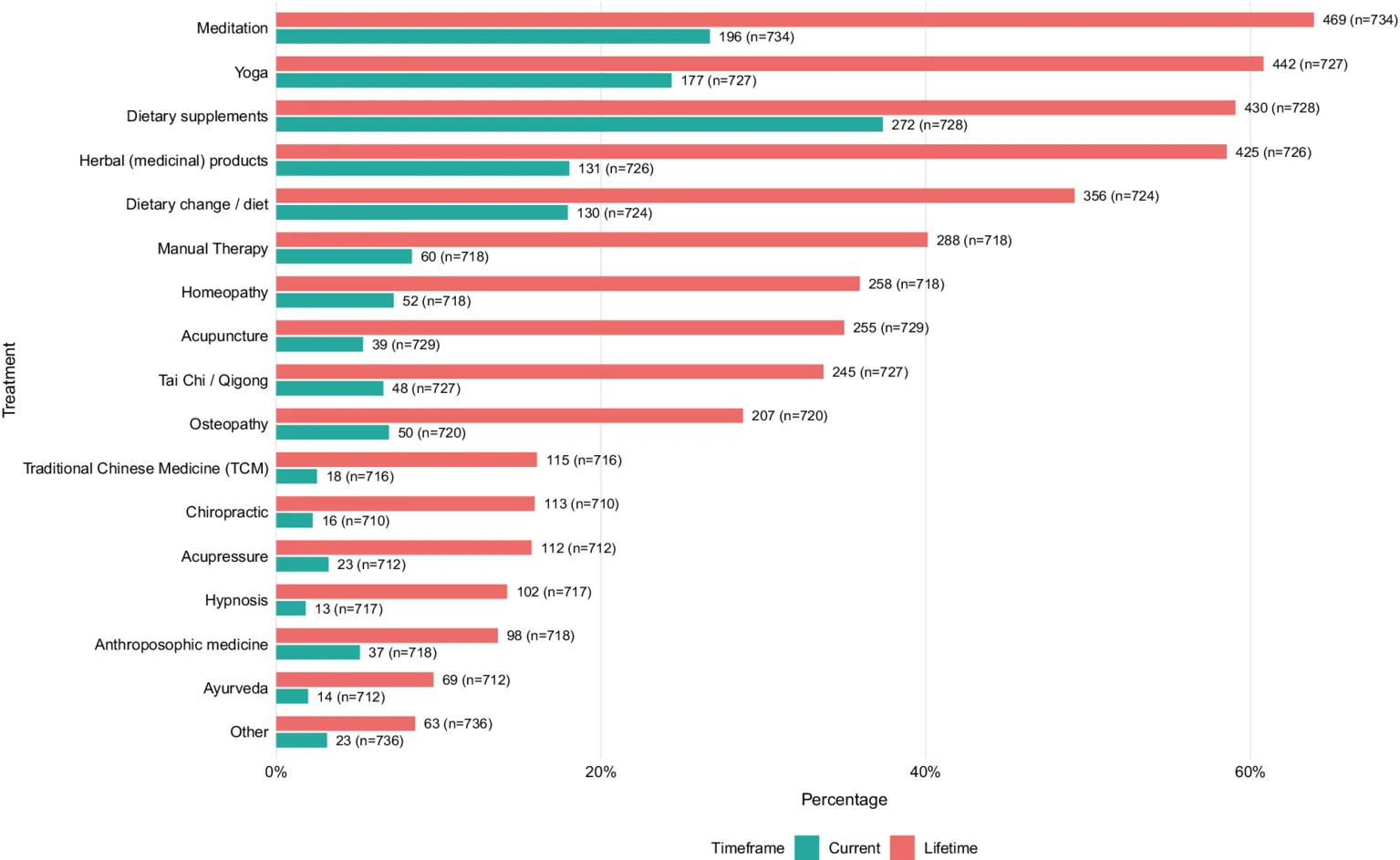
Lifetime use = proportion of participants who reported having used the CIM approach at any time during their lifetime (i.e., answered “yes” in at least one of the three timeframes: current, last 12 months, or ever).

Overall, 92.3% reported lifetime CIM use and 68.1% current use (Figure 1). Lifetime use was highest for meditation (63.9%), yoga (60.8%), dietary supplements (59.1%) and herbal remedies (58.5%). Moderate use was observed for dietary change (49.2%), manual therapy (40.1%), homeopathy (35.9%) and acupuncture (35.0%). Less frequent were Tai Chi/Qigong (33.7%), osteopathy (28.7%), chiropractic (15.9%), acupressure (15.7%), hypnosis (14.2%), anthroposophic medicine (13.6%) and Ayurveda (9.7%).

In open-text responses, dietary supplements were most frequently reported, including vitamin D, magnesium and B vitamins (particularly B12). The most common herbal remedies were St John’s wort (*Hypericum perforatum*), followed by valerian and ashwagandha. Additional products included lemon balm (*Melissa officinalis*), Bach flower remedies, herbal teas and cannabidiol (CBD). Participants also mentioned traditional approaches such as Kneipp hydrotherapy and movement therapy, as well as other CIM approaches including bioresonance, light therapy and Reiki.

### Perceived effects

Many participants reported positive experiences. The highest proportions were observed for meditation (76.9%) and yoga (74.6%). Positive ratings were also common for herbal medicinal products (58.4%) and dietary supplements (55.9%). Other interventions rated favourably included manual therapy (67.9%), Tai Chi/Qigong (64.4%), osteopathy (67.2%), acupuncture (50.4%) and homeopathy (48.2%). Dietary change showed 58.4% positive evaluations and chiropractic 62.3%. Hypnosis (53.1%) and anthroposophic medicine (74.0%) also showed high positive ratings despite lower use.

Some respondents reported no perceived benefit. “No benefit” was most frequent for homeopathy (34.9%), herbal medicinal products (31.2%) and dietary supplements (17.1%). Symptom worsening was rare but occurred most often with hypnosis (8.3%), dietary change (7.6%) and chiropractic treatment (6.4%).

Uncertainty (“do not know”) varied across CIM approaches, highest for dietary supplements (26.5%), acupuncture (19.1%) and Ayurveda (18.2%), suggesting limited experience or difficulty judging effectiveness (Table 2).

**Table 2:** Assessment of benefits by CIM-treatment use in lifetime use.

Treatments	Yes, helped	Yes, helped a little	No, did not help	No, worsening of symptoms	Do not know	Total (*n*)
Acupressure	*n* = 15 (13.9%)	*n* = 50 (46.3%)	*n* = 20 (18.5%)	*n* = 1 (0.9%)	*n* = 22 (20.4%)	108
Acupuncture	*n* = 41 (16.7%)	*n* = 83 (33.7%)	*n* = 68 (27.6%)	*n* = 7 (2.8%)	*n* = 47 (19.1%)	246
Tai Chi / Qi Gong	*n* = 50 (20.9%)	*n* = 104 (43.5%)	*n* = 47 (19.7%)	*n* = 1 (0.4%)	*n* = 37 (15.5%)	239
Traditional Chinese Medicine (TCM)	*n* = 29 (25.7%)	*n* = 43 (38.1%)	*n* = 22 (19.5%)	*n* = 1 (0.9%)	*n* = 18 (15.9%)	113
Osteopathy	*n* = 63 (31.8%)	*n* = 70 (35.4%)	*n* = 37 (18.7%)	*n* = 3 (1.5%)	*n* = 25 (12.6%)	198
Chiropractic	*n* = 31 (28.4%)	*n* = 37 (33.9%)	*n* = 24 (22.0%)	*n* = 7 (6.4%)	*n* = 10 (9.2%)	109
Manual Therapy	*n* = 80 (28.9%)	*n* = 108 (39.0%)	*n* = 53 (19.1%)	*n* = 6 (2.2%)	*n* = 30 (10.8%)	277
Dietary supplements	*n* = 81 (21.7%)	*n* = 128 (34.2%)	*n* = 64 (17.1%)	*n* = 2 (0.5%)	*n* = 99 (26.5%)	374
Dietary change / diet	*n* = 76 (22.1%)	*n* = 125 (36.3%)	*n* = 70 (20.3%)	*n* = 26 (7.6%)	*n* = 47 (13.7%)	344
Herbal (medicinal) remedies	*n* = 89 (22.2%)	*n* = 145 (36.2%)	*n* = 125 (31.2%)	*n* = 4 (1.0%)	*n* = 38 (9.5%)	401
Hypnosis	*n* = 24 (25.0%)	*n* = 27 (28.1%)	*n* = 30 (31.3%)	*n* = 8 (8.3%)	*n* = 7 (7.3%)	96
Yoga	n = 130 (30.7%)	*n* = 186 (43.9%)	*n* = 63 (14.9%)	*n* = 9 (2.1%)	*n* = 36 (8.5%)	424
Meditation	*n* = 153 (33.7%)	*n* = 196 (43.2%)	*n* = 60 (13.2%)	*n* = 16 (3.5%)	*n* = 29 (6.4%)	454
Homeopathy	*n* = 62 (24.9%)	*n* = 58 (23.3%)	*n* = 87 (34.9%)	*n* = 3 (1.2%)	*n* = 39 (15.7%)	249
Ayurveda	*n* = 18 (27.3%)	*n* = 25 (37.9%)	*n* = 11 (16.7%)	*n* = 0 (0.0%)	*n* = 12 (18.2%)	66
Anthroposophic medicine	*n* = 29 (30.2%)	*n* = 42 (43.8%)	*n* = 17 (17.7%)	*n* = 0 (0.0%)	*n* = 8 (8.3%)	96

### CIM use across primary psychiatric disorders

CIM use varied across primary psychiatric disorders (Table 3). Among participants with *depression* (*N* = 350), the most frequent approaches were meditation (64.0%), dietary supplements (56.3%), yoga (55.7%) and herbal remedies (53.4%), whereas Ayurveda (8.0%), chiropractic (12.3%) and hypnosis (11.4%) were rarely used.

**Table 3:** Most frequently used CIM treatments depending on the main psychiatric disorders (*n* = 662).

Characteristic (*n* = 662)	*n* (%)
Depression (*n* = 350)	
Meditation	224 (64.0%)
Dietary supplements	197 (56.3%)
Yoga	195 (55.7%)
Stress-related disorders (*n* = 124)	
Yoga	80 (64.5%)
Dietary supplements	74 (59.7%)
Herbal (medicinal) remedies	73 (58.9%)
Anxiety disorder (*n* = 82)	
Yoga	53 (64.6%)
Meditation	50 (61.0%)
Herbal (medicinal) remedies	47 (57.3%)
Personality disorder (*n* = 43)	
Meditation	29 (67.4%)
Dietary change/diet	26 (60.5%)
Herbal (medicinal) remedies	22 (51.2%)
Obsessive-compulsive disorder (*n* = 22)	
Herbal (medicinal) remedies	16 (72.7%)
Yoga	15 (68.2%)
Dietary supplements	12 (54.5%)
Somatic symptom disorder (*n* = 22)	
Manual Therapy	17 (77.3%)
Dietary supplements	14 (63.6%)
Acupuncture	13 (59.1%)
Eating disorder (*n* = 19)	
Herbal (medicinal) remedies	13 (68.4%)
Dietary supplements	13 (68.4%)
Dietary change / diet	13 (68.4%)

Among individuals reporting *stress-related disorders* (*N* = 124), the most common approaches were yoga (64.5%), dietary supplements (59.7%), herbal remedies (58.9%), dietary change (58.9%) and meditation (58.9%). Manual therapy (48.4%) and acupuncture (41.1%) were also frequently used, whereas anthroposophic medicine (8.9%) and Ayurveda (10.5%) were rare.

Among participants with *anxiety disorders* (*N* = 82), yoga (64.6%) and meditation (61.0%) were most frequent, followed by herbal remedies (57.3%) and dietary supplements (51.2%). Manual therapy (40.2%), Tai Chi/Qigong (35.4%) and acupuncture (34.1%) were also used, whereas hypnosis (14.6%) and Ayurveda (11.0%) were rare.

In *personality disorders* (*N* = 43), meditation (67.4%) and dietary change (60.5%) predominated. In *obsessive-compulsive disorder* (*N* = 22), herbal remedies (72.7%) and yoga (68.2%) were most common, while *somatic symptom disorders* (*N* = 22) showed high use of manual therapy (77.3%), dietary supplements (63.6%), and acupuncture (59.1%). *Eating disorders* (*N* = 19) were characterised by high use of herbal remedies, dietary supplements and dietary change (each 68.4%). Across diagnostic groups, meditation, yoga, dietary supplements and herbal remedies were consistently among the most frequently used CIM approaches.

### Physician awareness and sources of recommendation

**Figure 2:**
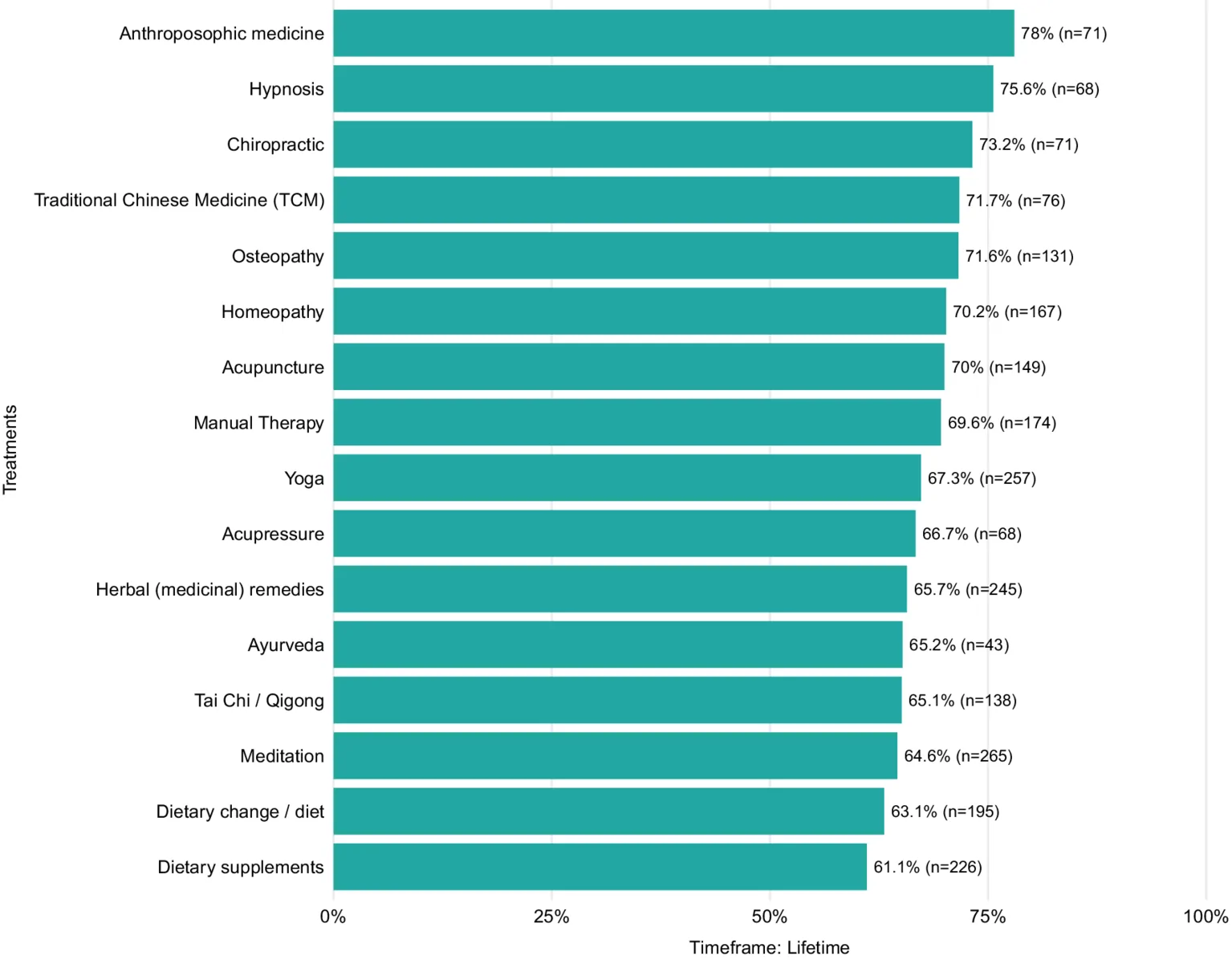
Horizontal bar chart showing the percentage of participants who reported that their physician was aware of their use of 15 CIM approaches. Anthroposophic medicine (78.0%), hypnosis (75.6%), and chiropractic (73.2%) had the highest levels of physician awareness, whereas dietary supplements (61.1%), dietary change (63.1%), and meditation (64.6%) had the lowest. The x-axis shows the percentage of participants reporting physician awareness, and the y-axis lists the CIM approaches. Each bar is labelled with the corresponding percentage and sample size (n).

Among CIM users, 60.9% reported that their physician was aware of this use, whereas 39.1% had not disclosed it. Physician awareness was similar across regions (Berlin 63.5%, Brandenburg 61.3%, other federal states 58.9%). Logistic regression adjusted for age and sex showed no significant regional differences (Brandenburg vs. Berlin: aOR 0.90, 95% CI 0.57–1.41; other federal states vs. Berlin: aOR 0.82, 95% CI 0.55–1.22); unadjusted analyses were similar. Disclosure varied by modality, highest for anthroposophic medicine (78.0%), hypnosis (75.6%) and chiropractic (73.2%) (Figure 2). CIM modality use did not differ meaningfully between those who disclosed use to their physician and those who did not (Supplementary Table 3).

The most frequent source of recommendation was self-directed information seeking, reported by 59.9% of users (internet searches 40.5%, guidebooks/manuals 19.4%). Recommendations from friends or acquaintances (31.2%) and physicians (27.7%) were also common, whereas psychotherapists (22.2%), alternative practitioners (14.4%) and pharmacists (4.0%) were less frequently cited.

Somatic-oriented therapies such as acupuncture, manual therapy, osteopathy and TCM were more often recommended by health care professionals, with about one-third reporting physician involvement (29–35%). In contrast, mind–body approaches including yoga, meditation, Tai Chi/Qigong and hypnosis were mainly initiated through informal networks or self-directed sources, with over 40% citing internet searches and 36–45% advice from friends (Supplementary Table 4).

### CIM effects and physician awareness

Among participants using CIM, physician awareness was associated with a more positive assessment of the effects of CIM. Participants whose physician was informed about their CIM use more frequently reported a positive assessment compared with those whose physician was not informed (92.0% vs. 81.1%). Conversely, non-positive assessments were more common among participants who had not informed their physician (18.9% vs. 8.0%). In logistic regression analyses adjusted for age and sex, physician awareness was significantly associated with higher odds of a positive assessment of CIM (adjusted aOR = 2.69, 95% CI 1.63–4.49, *p* < 0.001). Unadjusted analyses yielded comparable results.

## Discussion

In this sample of predominantly female outpatient individuals with psychiatric disorders, CIM use was highly prevalent, particularly accessible self-directed approaches such as meditation, yoga, herbal remedies, and dietary supplements. Physician involvement remained limited, as many individuals did not disclose CIM use despite its association with more positive evaluations when clinicians were aware.

### Strengths

To our knowledge, this study represents one of the first larger cross-sectional investigations of CIM use among individuals with psychiatric disorders in Germany. Recruitment covered a broad range of ambulatory mental health care settings, with psychiatric, psychosomatic and psychotherapeutic practices and day clinics in Berlin and Brandenburg randomly selected from the registry of the National Association of Statutory Health Insurance Physicians, strengthening external validity and reducing provider-level selection bias. A two-arm design combining a nationwide anonymous online survey with assisted on-site data collection increased reach and inclusivity. On-site support enabled participation of individuals with more severe illness or cognitive limitations who are often underrepresented in online-only surveys. Dissemination of the online survey was limited to general mental health organisations and self-help groups and did not target CIM-specific networks, reducing preferential recruitment of CIM-affine participants.

Standardised digital data collection via the SoSci Survey platform, hosted on secure institutional servers, minimised data entry errors and enabled plausibility and completeness checks.

### Limitations

The cross-sectional design precludes causal inference, and all associations should therefore be interpreted as non-causal. Data were based on self-reports and may be affected by recall and reporting bias, including the recruitment source variable. Selection bias cannot be excluded, as on-site recruitment was limited to Berlin and Brandenburg, the nationwide online arm relied on self-selected participants, inpatients were excluded, and the questionnaire was administered only in German, potentially underrepresenting individuals with limited German proficiency.

Although practices were randomly selected from the registry of the Association of Statutory Health Insurance Physicians, participation was voluntary and may have introduced centre-level selection effects. The high prevalence of CIM use may also partly reflect methodological and contextual influences such as online self-selection or the sociodemographic composition of the sample. Diagnostic status and medication use could not be externally verified, particularly in the anonymous online arm. Non-response bias cannot be ruled out, and anonymous data collection precluded post-hoc verification of individual responses. Furthermore, patient and service-user perspectives were not included in the content validation of the questionnaire, which may have limited its patient-centeredness.

### CIM use in psychiatric disorders: international context

International comparisons show substantial variation in CIM use among individuals with psychiatric disorders. In the World Mental Health Surveys across 25 countries, 3.6% of individuals with a 12-month mental disorder reported contact with a CIM provider, with higher rates in high-income countries (22).

In Norway, 17.8% of individuals with self-reported anxiety or depression had consulted a CIM provider, with few combining CIM with psychiatric outpatient care (25). Finnish data similarly indicate higher CIM use among individuals with comorbid anxiety and depression (31).

A U.S. survey likewise reported high CIM use among mental health consumers, often combined with conventional psychiatric care; more than half perceived CIM with medication as more effective than medication alone (32). German population surveys likewise report high lifetime CIM use (26).

In contrast, 68.1% of our sample reported current CIM use and 92.3% lifetime use, while 40.8% were taking psychopharmacological medication, indicating frequent use alongside standard treatment. The higher prevalence observed in our study may reflect combination with pharmacotherapy, predominantly self-initiated use, and methodological factors such as differences in CIM definitions, lifetime assessment and recruitment effects.

### Acceptance and controversy in CIM use

The high popularity of meditation and yoga in our sample aligns with international literature. Representative population data from Germany indicate widespread use and acceptance of CIM (26). Surveys among psychiatry researchers and clinicians likewise identify mind–body therapies such as meditation, yoga and hypnosis as promising interventions (21). In contrast, evaluations of homeopathy and hypnosis were more heterogeneous in our sample, reflecting ongoing controversies regarding their evidence base. Although used less frequently than mind–body approaches, a notable proportion of users reported limited or no benefit, and symptom worsening was most often reported for hypnosis. Population-based data from Sweden indicate that CIM use is largely driven by natural remedies, dietary supplements and self-help practices (33).

Overall, our findings highlight differing levels of acceptance across CIM approaches.

### Physician disclosure, recommendation sources, and patient evaluations

About 61% of participants reported informing their treating physicians about CIM use, with variation across approaches. In our sample, physician awareness was higher for approaches more closely embedded in therapeutic contexts. A systematic review reported wide variation in disclosure of CIM use to medical providers (7–80%); non-disclosure was commonly attributed to lack of provider inquiry, fear of disapproval, or the perception that disclosure was unnecessary (27).

The role of internet sources and advice from friends is consistent with survey data indicating the importance of informal information networks in CIM uptake (33).

Together with our findings, this suggests that physician awareness may be associated with patients’ evaluations of CIM effects. Prior literature likewise highlights the relevance of addressing complementary approaches within clinical care (34).

Moreover, surveys among psychiatry researchers and clinicians indicate heterogeneous perceptions of CIM approaches and a need for further training in this area (21). Evidence from U.S. surveys similarly indicates that non-disclosure of CIM use is widespread, with common reasons including lack of physician inquiry or the belief that disclosure is unnecessary (35). U.S. surveys further show that non-disclosure of CIM use is common and more likely when physician inquiry is absent, whereas disclosure increases with stronger patient–provider relationships (36). In our study, physician awareness of CIM use was associated with more favourable patient assessments, suggesting a potential role of routine clinical communication regarding CIM use.

### Clinical and research implications

Non-disclosure of CIM use was common, highlighting the importance of routine enquiry about CIM use in psychiatric care. The association between physician awareness and more positive patient evaluations of CIM may reflect communication-related or contextual factors, including stronger therapeutic relationships or greater perceived legitimacy of CIM approaches. Clinicians may particularly benefit from asking about commonly used CIM approaches such as herbal medicinal products, dietary supplements, meditation, and yoga, as these approaches were frequently reported in our sample and may otherwise remain undisclosed. This may be particularly relevant in outpatient psychiatric care, where CIM use frequently occurs alongside conventional treatment. Given potential interactions with psychopharmacological treatments (e.g., *Hypericum perforatum* with SSRIs), routine assessment, patient education and adequate physician competencies are essential.

Population-based survey data indicate that information on CIM is frequently obtained from media, the internet, and family or friends (33), highlighting the need for clinicians to address potentially unreliable information sources during consultations. Narrative reviews indicate the relevance of addressing complementary approaches within mental health care and suggest that improved integration and communication may support safer and more coordinated care (34), underscoring the need for adequate clinician knowledge and competencies to support informed discussions about CIM use.

Practical implications include routine assessment of commonly used CIM approaches, particularly herbal medicinal products and dietary supplements, to improve treatment coordination and identify potential interactions with psychopharmacological treatments.

Future longitudinal and interventional studies are required to clarify causal relationships and to evaluate the safety and effectiveness of frequently used modalities, particularly meditation, yoga, herbal medicinal products and dietary supplements. Additional priorities include CIM–medication interactions, determinants of disclosure and diagnosis-specific utilisation patterns.

Overall, CIM use appears to be a routine component of health-related self-management among individuals with psychiatric disorders. Given its high prevalence and incomplete disclosure, further rigorous clinical research is needed to evaluate the effectiveness and safety of CIM in mental disorders.

## Data Availability

The datasets generated and/or analyzed during the current study are available from the corresponding author upon reasonable request.

## References

[1] World Health Organization. Over a Billion People Living with Mental Health Conditions – Services Require Urgent Scale-Up. Geneva: World health Organization (2025).

[2] Fan Y Fan A Yang Z Fan D. Global burden of mental disorders in 204 countries and territories, 1990-2021: results from the global burden of disease study 2021. BMC Psychiatry. (2025) 25:486. doi: 10.1186/s12888-025-06932-y, 40375174 PMC12080068

[3] Casella CB Kousoulis AA Kohrt BA Bantjes J Kieling C Cuijpers P . Data gaps in prevalence rates of mental health conditions around the world: a retrospective analysis of nationally representative data. Lancet Glob Health. (2025) 13:e879–87. doi: 10.1016/S2214-109X(24)00563-1, 40288397

[4] Thom J Jonas B Reitzle L Mauz E Hölling H Schulz M. Trends in the diagnostic prevalence of mental disorders, 2012–2022: using Nationwide outpatient claims data for mental health surveillance. Dtsch Arztebl Int. (2024) 121:355. doi: 10.3238/arztebl.m2024.0052, 38686592 PMC11539879

[5] Walther L Junker S Rattay P Kuhnert R Hölling H Mauz E. Trends in depressive symptoms in Germany's adult population 2008-2023. Soc Psychiatry Psychiatr Epidemiol. (2025) 61:435–47. doi: 10.1007/s00127-025-02965-6 40696198 PMC12995932

[6] Adam N Neumann M Edelhäuser F. Patient satisfaction in inpatient psychiatric treatment compared with inpatient equivalent home treatment in Germany: an in-depth qualitative study. Front Health Serv. (2023) 3:1195614. doi: 10.3389/frhs.2023.1195614, 37457238 PMC10344693

[7] Ruggeri M Lasalvia A Bisoffi G Thornicroft G Vazquez-Barquero JL Becker T . Satisfaction with mental health services among people with schizophrenia in five European sites: results from the EPSILON study. Schizophr Bull. (2003) 29:229–45. doi: 10.1093/oxfordjournals.schbul.a007000, 14552499

[8] Kirkham EJ Fletcher-Watson S Beange I Chan SWY Lawrie SM. Patient satisfaction with mental and physical health services: findings from a UK-wide online survey. Wellcome Open Res. (2022) 7:198. doi: 10.12688/wellcomeopenres.17973.1, 37680686 PMC10480769

[9] Burstein O Shamir A Abramovitz N Doron R. Patients' attitudes toward conventional and herbal treatments for depression and anxiety: a cross-sectional Israeli survey. Int J Soc Psychiatry. (2022) 68:589–99. doi: 10.1177/0020764021992385, 33530827 PMC8938990

[10] Tomozei A. Zufriedenheit psychiatrischer Patienten mit der stationären Behandlung in einer psychiatrischen Fachklinik und der Psychiatrischen Klinik der LMU München (2006).

[11] Zhou Q Li X Yang D Xiong C Xiong Z. A comprehensive review and meta-analysis of neurological side effects related to second-generation antidepressants in individuals with major depressive disorder. Behav Brain Res. (2023) 447:114431. doi: 10.1016/j.bbr.2023.114431, 37044221

[12] Marks S. A clinical review of antidepressants, their sexual side-effects, post-SSRI sexual dysfunction, and serotonin syndrome. Br J Nurs. (2023) 32:678–82. doi: 10.12968/bjon.2023.32.14.678, 37495413

[13] Wen S Yan Y Shao J Xie H Wang M Dou Y . Comparative gastrointestinal effects of antidepressants for the acute treatment of adults with major depressive disorder: a network and dose–response meta-analysis. Transl Psychiatry. (2025) 16:34. doi: 10.1038/s41398-025-03751-3, 41290573 PMC12811249

[14] Hrabar V Studeniak T Pryima M Kondratskyi V. From efficacy to adversity: understanding the side effects of antidepressants - systematic review. Wiad Lek. (2025) 78:187–96. doi: 10.36740/WLek/197143, 40023872

[15] Singer S Maier L Paserat A Lang K Wirp B Kobes J . Wartezeiten auf einen Psychotherapieplatz vor und nach der Psychotherapiestrukturreform. Psychotherapeut. (2022) 67:176–84. doi: 10.1007/s00278-021-00551-0

[16] Wiegand HF Hölzel L Tüscher O Lieb K Falkai P Adorjan K. Mental health services in Germany–structures, outcomes and future challenges. Int Rev Psychiatry. (2025) 37:253–70. doi: 10.1080/09540261.2025.2479601, 40708412

[17] Sather EW Iversen VC Svindseth MF Crawford P Vasset F. Exploring sustainable care pathways-a scoping review. BMC Health Serv Res. (2022) 22:1595. doi: 10.1186/s12913-022-08863-w, 36585672 PMC9801530

[18] World Health Organization. Traditional, Complementary and Integrative Medicine. (2026). Geneva: World Health Organization. Available online at: https://www.who.int/health-topics/traditional-complementary-and-integrative-medicine? (Accessed June 22, 2026)

[19] Pellegrini N Ruggeri M. The diffusion and the reason for the use of complementary and alternative medicine among users of mental health services: a systematic review of literature. Epidemiol Psichiatr Soc. (2007) 16:35–49. doi: 10.1017/S1121189X00004590, 17427603

[20] Zare K Hedayati A Hashemi Y Ostovari Z Naghizadeh MM Hashempur MH . A cross-sectional study on the use of complementary and alternative medicine in insomnia patients referred to psychiatric clinics in Iran. Sleep Breath. (2025) 29:192. doi: 10.1007/s11325-025-03354-8, 40402334

[21] Ng JY Kochhar J Cramer H. An international, cross-sectional survey of psychiatry researchers and clinicians: perceptions of complementary, alternative, and integrative medicine. Front Psych. (2024) 15:1416803. doi: 10.3389/fpsyt.2024.1416803, 39205853 PMC11349733

[22] de Jonge P Wardenaar KJ Hoenders HR Evans-Lacko S Kovess-Masfety V Aguilar-Gaxiola S . Complementary and alternative medicine contacts by persons with mental disorders in 25 countries: results from the world mental health surveys. Epidemiol Psychiatr Sci. (2018) 27:552–67. doi: 10.1017/S2045796017000774, 29283080 PMC6849470

[23] Ashraf H Salehi A Sousani M Sharifi MH. Use of complementary alternative medicine and the associated factors among patients with depression. Evid Based Complement Alternat Med. (2021) 2021:1–8. doi: 10.1155/2021/6626394, 33854557 PMC8019377

[24] Wemrell M Olsson A Landgren K. The use of complementary and alternative medicine (CAM) in psychiatric units in Sweden. Issues Ment Health Nurs. (2020) 41:946–57. doi: 10.1080/01612840.2020.1744203, 32497455

[25] Hansen AH Kristoffersen AE. The use of CAM providers and psychiatric outpatient services in people with anxiety/depression: a cross-sectional survey. BMC Complement Altern Med. (2016) 16:461. doi: 10.1186/s12906-016-1446-9, 27835971 PMC5106802

[26] Jeitler M Ortiz M Brinkhaus B Sigl M Hoffmann R Trübner M . Use and acceptance of traditional, complementary and integrative medicine in Germany-an online representative cross-sectional study. Front Med (Lausanne). (2024) 11:1372924. doi: 10.3389/fmed.2024.1372924 38545512 PMC10965565

[27] Foley H Steel A Cramer H Wardle J Adams J. Disclosure of complementary medicine use to medical providers: a systematic review and meta-analysis. Sci Rep. (2019) 9:1573. doi: 10.1038/s41598-018-38279-8, 30733573 PMC6367405

[28] Davis EL Oh B Butow PN Mullan BA Clarke S. Cancer patient disclosure and patient-doctor communication of complementary and alternative medicine use: a systematic review. Oncologist. (2012) 17:1475–81. doi: 10.1634/theoncologist.2012-0223, 22933591 PMC3500370

[29] Siewert J Wackermann H Brinkhaus B Teut M Jansen E. Experiences with complementary and integrative medicine in mental healthcare: a qualitative substudy of the PSYKIM project. Front Med (Lausanne). (2025) 12:1629436. doi: 10.3389/fmed.2025.1629436, 40963583 PMC12436651

[30] World Health Organization. (2004) The global burden of dieseas. Available online at: https://apps.who.int/iris/bitstream/handle/10665/43942/9789241563710_eng.pdf?sequence=1&isAllowed=y (Accessed June 22, 2026).

[31] Wahlström M Sihvo S Haukkala A Kiviruusu O Pirkola S Isometsä E. Use of mental health services and complementary and alternative medicine in persons with common mental disorders. Acta Psychiatr Scand. (2008) 118:73–80. doi: 10.1111/j.1600-0447.2008.01192.x, 18595177

[32] Clossey L DiLauro MD Edwards JP Hu C Pazaki H Monge A . Complementary and alternative medicine (CAM) use among mental health consumers. Community Ment Health J. (2023) 59:1549–59. doi: 10.1007/s10597-023-01142-w, 37261659

[33] Danell JB Wode K Jong M Kristoffersen AE van der Werf E Nordberg JH. Use of complementary and alternative medicine (CAM) in Sweden: a cross-sectional survey. Scand J Prim Health Care. (2025) 43:529–37. doi: 10.1080/02813432.2025.2466180, 39988756 PMC12377132

[34] Gureje O Nortje G Makanjuola V Oladeji BD Seedat S Jenkins R. The role of global traditional and complementary systems of medicine in treating mental health problems. Lancet Psychiatry. (2015) 2:168–77. doi: 10.1016/S2215-0366(15)00013-9 26359753 PMC4456435

[35] Jou J Johnson PJ. Nondisclosure of complementary and alternative medicine use to primary care physicians: findings from the 2012 National Health Interview Survey. JAMA Intern Med. (2016) 176:545–6. doi: 10.1001/jamainternmed.2015.8593, 26999670

[36] Faith J Thorburn S Tippens KM. Examining CAM use disclosure using the behavioral model of health services use. Complement Ther Med. (2013) 21:501–8. doi: 10.1016/j.ctim.2013.08.002, 24050587

