# Supplementary material for: Use of complementary and integrative medicine for mental disorders–a cross-sectional study in Germany
Source: Front Med (Lausanne). 2026 Aug 19;13:1882619. doi: 10.3389/fmed.2026.1882619 (PMC13534134; doi:10.3389/fmed.2026.1882619)
Supplement: Supplementary file 1 [file Supplementary_file_1.docx]

**Supplementary Material 1**

**PSYKIM Survey Instrument (English Translation)**

The original questionnaire was administered in German. The following English translation is provided for transparency and documentation of the survey items used in the PSYKIM study.

The questionnaire assessed psychiatric diagnoses, use of complementary and integrative medicine (CIM), perceived effects, disclosure to physicians, sources of recommendation, and sociodemographic characteristics.

**Section 1: Psychiatric Condition**

**1. What is your main psychiatric or psychosomatic diagnosis?**

- Depression
- Other mood disorder (e.g., bipolar disorder, mania)
- Anxiety disorder (e.g., panic disorder, generalized anxiety disorder, phobias)
- Eating disorder (e.g., anorexia nervosa, bulimia nervosa, binge-eating disorder)
- Personality disorder (e.g., borderline personality disorder)
- Schizophrenia
- Other psychotic disorder
- Substance use disorder (alcohol or drugs)
- Obsessive–compulsive disorder
- Somatic symptom disorder
- Stress-related disorders (e.g., PTSD, adjustment disorder)
- Dementia
- Other psychiatric diagnosis: ______
- I have a psychiatric condition but do not know the diagnosis

**2. Do you have additional psychiatric diagnoses?**

- Yes
- No

If yes, which ones? (multiple answers possible)

- Depression
- Other mood disorder (e.g., bipolar disorder, mania)
- Anxiety disorder
- Personality disorder
- Eating disorder
- Schizophrenia
- Other Psychotic disorder
- Substance use disorder
- Obsessive–compulsive disorder
- Somatic symptom disorder
- Stress-related disorders
- Dementia
- Other diagnosis: ______
- I have a psychiatric condition but do not know the diagnosis

**3. Duration of your main psychiatric condition**

- Less than 6 months
- More than 6 months
- Currently no symptoms

**4. Course of illness**

- First occurrence
- Recurrent

**5. Have you used digital therapies (apps or online programs) for your condition?**

- Yes
- No

**Section 2: Sociodemographic Characteristics**

**1. Gender**

- Female
- Male
- Diverse

**2. Age**

(open numeric response)

**3. Federal state of residence**

- Berlin
- Brandenburg
- Other German federal state

**4. Participation mode**

- Online survey
- Survey in outpatient clinic / practice / day clinic

**5. Highest educational level**

- No vocational training
- Vocational training
- Vocational school
- Technical qualification (e.g., master craftsman, technician)
- University of applied sciences degree
- University degree
- Currently in education/training
- Other qualification

**6. Current employment status**

- Employed
- Self-employed
- Unemployed
- Student
- Retired
- Other

**7. Are you currently on sick leave due to your psychiatric condition?**

- Yes
- No

**8. Are you receiving disability pension due to your psychiatric condition?**

- Yes
- No

**9. Living situation**

- Living alone
- Living with others in the same household

**10. Health insurance**

- Statutory health insurance
- Private health insurance
- Other insurance
- No insurance

**Section 3: Use of Complementary and Integrative Medicine (CIM)**

Participants were asked whether they had **ever used**, **currently used**, or **used within the past 12 months** the following CIM approaches for their psychiatric symptoms.

For each therapy, perceived effects were rated as:

- Complete improvement
- Partial improvement
- No improvement
- Worsening of symptoms
- Uncertain

**11. CIM modalities assessed**

1. Acupuncture
2. Acupressure
3. Traditional Chinese Medicine
4. Tai Chi / Qigong
5. Osteopathy
6. Chiropractic treatment
7. Manual therapy
8. Herbal medicinal products
9. Dietary supplements
10. Dietary change / special diet
11. Hypnosis
12. Yoga
13. Meditation
14. Homeopathy
15. Ayurveda
16. Anthroposophic medicine
17. Other complementary therapies (free text)

Participants reporting herbal medicines or dietary supplements were asked to list the specific products used.

**Section 4: Physician Awareness and Recommendations**

Displayed only if participants reported CIM use.

**12. Is your treating physician aware of your use of complementary therapies?**

- Yes
- No

**13. Who recommended the complementary therapy?**
(multiple responses possible)

- Physician
- Psychotherapist
- Alternative practitioner
- Pharmacist
- Internet research
- Friends or acquaintances

**14. Have you used conventional medications for your psychiatric condition?**

- Currently
- Within the past 12 months
- Previously
- Never

If yes, participants could report medications in free text.

**Section 5: Economic Aspects**

**15. Did you pay privately for complementary therapies?**

- Yes
- No

**16. Approximate monthly expenses for complementary therapies**

- Up to €50
- €51–100
- €101–200
- More than €200

**17. Monthly disposable income after fixed expenses**

- ≤ €50
- €51–100
- €101–200
- €201–500
- €501–1000
- €1001–1500
- €1500

**Section 6: Health-Related Quality of Life**

Health-related quality of life was assessed using the **EQ-5D-5L instrument**, covering:

- Mobility
- Self-care
- Usual activities
- Pain/discomfort
- Anxiety/depression

Each dimension was rated on a **five-level scale** (no problems to extreme problems).

Participants also rated their **current health status on a visual analogue scale (0–100)**.
