# Supplementary material for: Use of complementary and integrative medicine for mental disorders–a cross-sectional study in Germany
Source: Front Med (Lausanne). 2026 Aug 19;13:1882619. doi: 10.3389/fmed.2026.1882619 (PMC13534134; doi:10.3389/fmed.2026.1882619)
Supplement: Supplementary file 2 [file Table_1.docx]

*Supplementary Table 1. Primary psychiatric disorder (N = 768)*

| Psychiatric disorder (N = 768) | n (%) |
| --- | --- |
| Depression | 350 (45.6%) |
| Other mood disorder | 27 (3.5%) |
| Anxiety disorder | 82 (10.7%) |
| Eating disorder | 19 (2.5%) |
| Personality disorder | 43 (5.6%) |
| Schizophrenia | 17 (2.2%) |
| Other psychotic or delusional disorders | 4 (0.5%) |
| Substance use disorder (alcohol/drugs) | 12 (1.6%) |
| Nicotine dependence | 3 (0.4%) |
| Obsessive–compulsive disorder | 22 (2.9%) |
| Somatic symptom disorder | 22 (2.9%) |
| Stress-related disorders | 124 (16.2%) |
| Dementia | 2 (0.3%) |
| Other psychiatric diagnosis | 4 (0.5%) |
| Unknown psychiatric diagnosis | 9 (1.2%) |
| Attention-deficit / hyperactivity disorder (ADHD) | 13 (1.7%) |
| Autism spectrum disorder (ASD) | 4 (0.5%) |
| Mixed anxiety and depressive disorder | 10 (1.3%) |
| Not reported | n =1 |
