# Supplementary material for: Use of complementary and integrative medicine for mental disorders–a cross-sectional study in Germany
Source: Front Med (Lausanne). 2026 Aug 19;13:1882619. doi: 10.3389/fmed.2026.1882619 (PMC13534134; doi:10.3389/fmed.2026.1882619)
Supplement: Supplementary file 3 [file Table_2.docx]

#### Supplementary Table 2. Additional psychiatric disorder

#### Other psychiatric disorder (percentages refer only to participants who reported additional psychiatric disorders; N = 491)

| Psychiatric disorder (N = 491) | n (%) |
| --- | --- |
| Depression | 204 (41.5%) |
| Other mood disorder | 21 (4.3%) |
| Anxiety disorder | 184 (37.5%) |
| Eating disorder | 68 (13.8%) |
| Personality disorder | 58 (11.8%) |
| Schizophrenia | 8 (1.6%) |
| Other psychotic or delusional disorders | 11 (2.2%) |
| Substance use disorder (alcohol/drugs) | 44 (9.0%) |
| Nicotine dependence | 29 (5.9%) |
| Obsessive–compulsive disorder | 56 (11.4%) |
| Somatic symptom disorder | 98 (20.0%) |
| Stress-related disorders | 119 (24.2%) |
| Dementia | 0 (0.0%) |
| Other psychiatric diagnosis | 11 (2.2%) |
| Unknown psychiatric diagnosis | 4 (0.8%) |
| Attention-deficit / hyperactivity disorder (ADHD) | 21 (4.3%) |
| Autism spectrum disorder (ASD) | 2 (0.4%) |
| Mixed anxiety and depressive disorder | 0 (0.0%) |
