# Supplementary material for: Use of complementary and integrative medicine for mental disorders–a cross-sectional study in Germany
Source: Front Med (Lausanne). 2026 Aug 19;13:1882619. doi: 10.3389/fmed.2026.1882619 (PMC13534134; doi:10.3389/fmed.2026.1882619)
Supplement: Supplementary file 4 [file Table_3.docx]

*Supplementary Table 3: Use of CIM Modalities by Physician Disclosure Status*

| Treatments | Physician informed (n = 363) n (%) | Physician not informed (n = 233) n (%) |
| --- | --- | --- |
| Meditation | 265 (73.0) | 145 (62.2) |
| Yoga | 257 (70.8) | 125 (53.6) |
| Herbal (medicinal) remedies | 245 (67.5) | 128 (54.9) |
| Dietary supplements | 226 (62.3) | 144 (61.8) |
| Dietary change / diet | 195 (53.7) | 114 (48.9) |
| Manual therapy | 174 (47.9) | 76 (32.6) |
| Homeopathy | 167 (46.0) | 71 (30.5) |
| Acupuncture | 149 (41.0) | 64 (27.5) |
| Tai Chi / Qigong | 138 (38.0) | 74 (31.8) |
| Osteopathy | 131 (36.1) | 52 (22.3) |
| Traditional Chinese Medicine (TCM) | 76 (20.9) | 30 (12.9) |
| Chiropractic | 71 (19.6) | 26 (11.2) |
| Anthroposophic medicine | 71 (19.6) | 20 (8.6) |
| Acupressure | 68 (18.7) | 34 (14.6) |
| Hypnosis | 68 (18.7) | 22 (9.4) |
| Ayurveda | 43 (11.8) | 23 (9.9) |

Percentages refer to the proportion of users within each disclosure group. Multiple responses were possible.
