# Supplementary material for: Use of complementary and integrative medicine for mental disorders–a cross-sectional study in Germany
Source: Front Med (Lausanne). 2026 Aug 19;13:1882619. doi: 10.3389/fmed.2026.1882619 (PMC13534134; doi:10.3389/fmed.2026.1882619)
Supplement: Supplementary file 5 [file Table_4.docx]

Supplementary Table 4. Source of Recommendation and use of CIM

| Treatments | Doctor | Friends | Psycho-therapist | Alternative practitioner | Pharmacist | Internet | Advisor |
| --- | --- | --- | --- | --- | --- | --- | --- |
| Acupressure | n=34 (30.4%) | n=40 (35.7%) | n=32 (28.6%) | n=26 (23.2%) | n=9 (8.0%) | n=50 (44.6%) | n=29 (25.9%) |
| Acupuncture | n=69 (27.1%) | n=77 (30.2%) | n=48 (18.8%) | n=56 (22.0%) | n=9 (3.5%) | n=77 (30.2%) | n=51 (20.0%) |
| Anthroposophic medicine | n=43 (43.9%) | n=36 (36.7%) | n=32 (32.7%) | n=31 (31.6%) | n=5 (5.1%) | n=32 (32.7%) | n=24 (24.5%) |
| Ayurveda | n=21 (30.4%) | n=32 (46.4%) | n=17 (24.6%) | n=24 (34.8%) | n=2 (2.9%) | n=39 (59.1%) | n=23 (33.3%) |
| Chiropractic | n=29 (25.7%) | n=40 (35.4%) | n=23 (20.4%) | n=23 (20.4%) | n=7 (6.2%) | n=43 (38.1%) | n=27 (23.9%) |
| Dietary change/ diet | n=92 (25.8%) | n=103 (28.9%) | n=73 (20.5%) | n=57 (16.0%) | n=12 (3.4%) | n=149 (41.9%) | n=68 (19.1%) |
| Dietary supplements | n=110 (25.6%) | n=124 (28.8%) | n=82 (19.1%) | n=67 (15.6%) | n=19 (4.4%) | n=173 (40.2%) | n=89 (20.7%) |
| Herbal (medicinal) remedies | n=114 (26.8%) | n=131 (30.8%) | n=77 (18.1%) | n=71 (16.7%) | n=24 (5.6%) | n=170 (40.0%) | n=91 (21.4%) |
| Homeopathy | n=78 (30.2%) | n=101 (39.1%) | n=59 (22.9%) | n=67 (26.0%) | n=17 (6.6%) | n=113 (43.8%) | n=57 (22.1%) |
| Hypnosis | n=24 (23.5%) | n=41 (40.2%) | n=21 (20.6%) | n=22 (21.6%) | n=6 (5.9%) | n=46 (45.1%) | n=26 (25.5%) |
| Manual Therapy | n=81 (28.1%) | n=90 (31.2%) | n=66 (22.9%) | n=52 (18.1%) | n=13 (4.5%) | n=105 (36.5%) | n=54 (18.8%) |
| Meditation | n=119 (25.4%) | n=149 (31.8%) | n=104 (22.2%) | n=69 (14.7%) | n=17 (3.6%) | n=187 (39.9%) | n=91 (19.4%) |
| Osteopathy | n=63 (30.4%) | n=67 (32.4%) | n=46 (22.2%) | n=50 (24.2%) | n=13 (6.3%) | n=82 (39.6%) | n=52 (25.1%) |
| Tai Chi / Qigong | n=68 (27.8%) | n=80 (32.7%) | n=57 (23.3%) | n=42 (17.1%) | n=13 (5.3%) | n=89 (36.3%) | n=59 (24.1%) |
| Traditional Chinese Medicine (TCM) | n=38 (33.0%) | n=43 (37.4%) | n=28 (24.3%) | n=36 (31.3%) | n=5 (4.3%) | n=46 (40.0%) | n=34 (29.6%) |
| Yoga | n=115 (26.0%) | n=141 (31.9%) | n=100 (22.6%) | n=63 (14.3%) | n=12 (2.7%) | n=171 (38.7%) | n=86 (19.5%) |
